# The relationship between self‐compassion and caring behaviour in nurses: A cross‐sectional study

**DOI:** 10.1111/inr.13017

**Published:** 2024-07-05

**Authors:** Arif Özparlak, Dudu Karakaya, Hilal Kara, Esra Çelik

**Affiliations:** ^1^ Department of Psychiatric Nursing Akdeniz University Faculty of Nursing Antalya Turkey; ^2^ Ministry of Health Korkuteli Public Hospital Intensive Care Unit Antalya Turkey

**Keywords:** Caring behaviour, cross‐sectional study, nursing, self‐compassion

## Abstract

**Aim:**

To examine the relationship between self‐compassion and caring behaviour in nurses.

**Background:**

Self‐compassion can influence nurses’ ability to cope with stress and their job performance. High levels of self‐compassion may play a role in nurses’ coping with compassion fatigue and burnout. This may make the concept of self‐compassion in nurses an important variable for effective care.

**Methods:**

This is a cross‐sectional study. Study data were collected between March and May 2022 from 331 nurses at a hospital in the city of Antalya, Turkey. A personal information form, the Self‐Compassion Scale (SCS), and the Caring Behaviour Inventory‐24 (CBI‐24) were used to collect data, and the program SPSS 23.0 was used in data evaluation. Descriptive statistical methods, Pearson correlation analysis and multiple linear regression analysis were used in the analysis of data. The STROBE checklist was followed for this cross‐sectional study.

**Results:**

The nurses’ mean scores were 3.50 ± 0.61 on SCS and 5.21 ± 0.56 on CBI‐24. A positive correlation was found between the nurses’ self‐compassion levels and caring behaviour. Also, the SCS sub‐dimension of mindfulness, working in intensive care and working willingly in the nursing profession significantly predicted caring behaviour. These variables explain 19.4% of the variance of caring behaviour.

**Conclusions:**

The nurses’ self‐compassion levels were medium and their caring behaviour was at a high level, and caring behaviour was higher in those who worked in intensive care, those who were working willingly in the nursing profession, and in those with high scores on the self‐compassion sub‐dimension of mindfulness.

**Implications for nursing and health policy:**

It is important to strengthen nurses’ self‐compassion skills to develop their caring behaviour. In particular, giving nurses in clinics mindfulness‐based education will help them to increase their awareness concerning their own lives and to develop their caring behaviour.

## INTRODUCTION

In the field of nursing, caring is accepted as a basic element of nursing interventions (Dahlke & Stahlke, 2017). Caring is an interpersonal process characterised by nurses’ professional knowledge, skills and personal maturity, resulting in the protection of patients, providing emotional support and meeting their biopsychosocial needs (Drahošová & Jarošová, 2016; Liao et al., 2023). All kinds of actions that nurses display to achieve the process of caring are defined as caring behaviour (Leyva et al., 2015). Caring behaviour includes such things as being prepared for caring, being honest, listening attentively, giving treatments correctly and on time, being individual‐centred and taking cultural differences into account (Watson, 2012).

Self‐compassion is one of the important factors that can affect caring behaviour (Alquwez et al., 2021); it expresses mindfulness of a person's own feelings and being affectionate and kind to oneself when negative situations are encountered (Neff & Tirch, 2013). Persons with high self‐compassion have a less judgemental attitude towards others and accept negative experiences as a natural part of human life (Marshall & Brockman, 2016). Recently, self‐compassion has become an important concept in helping nurses to cope with difficulties and providing protection from these difficulties (Andrews et al., 2020). Nurses with high self‐compassion have higher coping skills, self‐respect and stability (Joy et al., 2023). Nurses with low stress levels and high coping skills have improved work performance and caring qualities (Stab et al., 2016). Self‐compassion can also play a role in how nurses cope with compassion fatigue and exhaustion (Dev et al., 2020). This may make the concept of self‐compassion an important variable for effective care in nurses. In order for nurses to provide effective care, it is necessary for them to behave compassionately to themselves (Durkin et al., 2016).

## BACKGROUND

In the literature, nurses’ self‐compassion scores were found to be medium (Efil et al., 2022; Kibret et al., 2022), while their caring behaviour scores were seen to be high (Atharyan et al., 2018; Duarte et al., 2016; Malik et al., 2022). Additionally, studies have found male gender (Ilkafah et al., 2023), greater age and work experience (Arsat et al., 2023), working in intensive care (Erol & Turk, 2019), being married (Lee & Seo, 2022), and higher education levels (Fradelos et al., 2022) to be correlated with caring behaviour. However, some studies have reported that variables such as age (Kibret et al., 2022), gender, educational status (Lee & Seo, 2022) and marital status (Fallah et al., 2023) were not found to be correlated with caring behaviour.

Few studies were seen in the literature examining the relationship between self‐compassion and caring behaviour in nurses. In a study by Alquwez et al. (2021) with nursing students, it was found that the self‐compassion sub‐dimensions of self‐kindness and common humanity significantly predicted caring behaviour. It was also stated in this study that the caring behaviour levels of students with an extended family and of intern students were higher (Alquwez et al., 2021). In a study by Varghese (2020), a positive correlation was found in psychiatry nurses between self‐compassion levels and perceived caring efficacy. The researchers stressed that more research needed to be conducted with nurses working in different cultures and different fields in order to clarify the relationship between self‐compassion and caring behaviour (Altun et al., 2020; Varghese, 2020). It is thought that the determination of the relationship between self‐compassion and caring behaviour in nurses is important in order to provide effective care to patients and to increase patient satisfaction. It is expected that study results will provide data that will improve the caring behaviour of nurses. The aim of this research was to determine the levels of caring behaviour and self‐compassion in nurses, to examine the relationship between caring behaviour and self‐compassion, and to determine the variables predicting caring behaviour. With this aim, the research questions of the study are as follows: What are the levels of self‐compassion and caring behaviour among nurses? Is there a relationship between self‐compassion and caring behaviour in nurses? Do nurses' sociodemographic characteristics, work characteristics, levels of self‐compassion and status of receiving training on self‐compassion predict caring behaviours?

## METHODS

### Design and participants

This was a cross‐sectional study. Data collection was performed between March and May 2022 at a hospital in the city of Antalya, Turkey. The study universe was composed of individuals employed as nurses at Akdeniz University Hospital. The participants were identified using convenience sampling method. The participants were nurses working in clinical units such as internal medicine, obstetrics and gynaecology and psychiatry, outpatient units such as internal medicine, obstetrics and gynaecology and psychiatry, adult or paediatric intensive care and emergency department units. The sample size was calculated using the program G*Power version 3.1. No similar study with the same sample group was found in the literature, so Cohen's mean effect size (0.15) was used (Cohen, 1988). The sample size was found to be a minimum of 230 at a power of 95%, accepting Type 1 error of 0.05, Type 2 error of 0.05 and effect size of 0.15 from a multiple linear regression model. As a result, the study was completed with 331 nurses. This study was reported in line with the Strengthening the Reporting of Observational studies in Epidemiology (STROBE), which is used in cross‐sectional studies (Supplementary Material S1) (von Elm et al., 2008).

### Materials and procedure

A personal information form, the Self‐Compassion Scale (SCS), and the Caring Behaviour Inventory‐24 (CBI‐24) were used to collect data. First, a list was obtained of all of the units of the institution where data were to be collected, and the number of nurses working in these units was found. After that, all units were visited by the researchers, and the nurses were given information on the aims and methods of the research. Nurses who agreed to participate in the study completed the data collection forms. This took approximately 15 minutes.

### Personal information form

The form was developed by the researchers in line with the literature (Kibret et al., 2022; Varghese, 2020), and consisted of nine questions, asking about sociodemographic information such as the nurses' age, gender and marital status, their work characteristics and whether they had received education on self‐compassion.

### Self‐compassion scale

The SCS was developed by Neff (2003), and the adaptation to Turkish was performed by Akın et al. (2007). The scale consists of a total of 26 items, with sub‐dimensions of self‐kindness, self‐judgement, common humanity, isolation, mindfulness and over‐identification. Self‐kindness means having a kind and gentle approach to oneself; self‐judgement means judging oneself harshly in the face of lack of success; common humanity means accepting that everyone can have negative experiences; isolation means thinking that negative experiences only happen to oneself and isolating oneself from other people; mindfulness means being aware of and accepting negative experiences, and over‐identification means getting caught up in negative experiences and feelings. The scale is of five‐point Likert type, and the participants are asked to mark one of the choices of ‘never’, ‘seldom’, ‘often’, ‘usually’ or ‘always’. Each sub‐dimension is scored separately, and a total self‐compassion mean is also obtained. The total score is calculated by adding together the scale items and dividing by 26. A total score obtained from a scale of 1–2.49 shows a low level of self‐compassion, 2.5–3.5 a medium level and 3.51–5 a high level. The Cronbach alpha values of the scale's sub‐dimensions vary from 0.72 to 0.80 (Akın et al., 2007). In this study, the overall Cronbach alpha value was calculated as 0.93.

### Caring behaviour inventory‐24

CBI‐24 was developed by Wu et al. (2006), and the Turkish validity and reliability work was performed by Kurşun and Kanan (2012). The scale has 24 items in four sub‐dimensions of assurance, knowledge and skill, respectfulness and connectedness. The assurance dimension covers administering the patient's treatments on time and alleviating symptoms; the knowledge and skill dimension covers correct administration of treatment and follow‐up; the dimension of respectfulness covers approaching the patient as an individual and establishing empathy, and the connectedness dimension covers behaving patiently, being understanding and including the patient in care. The CBI‐24 is a six‐point Likert‐type scale. As the scores of the sub‐dimensions and the total score increase, the level of perceived care quality also increases. The scores of the scale items are added together and then divided by 24, giving a score of between 1 and 6. The Cronbach's alpha value of the Turkish version of the CBI‐24 was found to be 0.96 (Kurşun & Kanan, 2012). In this study, the overall Cronbach alpha value was calculated as 0.95.

### Ethical considerations

Permission to use the scales used in the study was obtained by email from the relevant authors. Ethics committee approval (Approval No: 70904504/612) was obtained from the Akdeniz University Clinical Research Ethics Committee. Also, information on the research was given orally to the nurses participating in the study, and their individual written consent was obtained.

### Data analysis

Data were assessed with the program SPSS 23.0 (Statistical Package for Social Sciences for Windows). Before beginning analysis, the data were examined for missing values, outliers, normalcy, collinearity and the problem of multicollinearity. To determine whether data conformed to normal distribution, the kurtosis and skewness coefficients and histogram graphs of the total scores of the scales were examined. Also, Kolmogorov–Smirnov and Shapiro–Wilk tests were performed. In the kurtosis and skewness coefficients, ± 1 was accepted as the normal limit (Tabachnick & Fidell, 2012). To evaluate the nurses’ demographic data in the study, numerical values, percentages and means were used. To determine the relationships between SCS and CBI‐24, Pearson correlation analysis was used, and multiple linear regression analysis was used to determine the predictors of CBI‐24. In statistical decisions, the level of *p* < 0.05 was accepted as a significant difference.

## RESULTS

### Demographic characteristics

It was found that 90.6% (*n* = 300) of the nurses were female, 62.8% (*n* = 208) were married, 81% (*n* = 268) had bachelor's degrees and 36.9% (*n* = 122) were aged between 20 and 29 years. While 54.4% (*n* = 180) of the nurses were working in the clinics, 29.6% (*n* = 98) had less than five years of experience. Finally, 91.5% (*n* = 303) of the participants stated that they were working of their own free will in the unit where they were working, 79.8% (*n* = 264) stated that they had chosen the nursing profession willingly, and 84.3% (*n* = 279) stated that they had had no education on self‐compassion (Table 1).

**TABLE 1:** Descriptive characteristics of nurses (*n* = 331).

Variables	*n*	%
Age		
20–29	122	36.9
30–39	113	34.1
>40	96	29
Gender		
Female	300	90.6
Male	31	9.4
Educational level		
High school	12	3.6
Associate degree	15	4.5
Bachelor's degree	268	81
Postgraduate degree	36	10.9
Marital status		
Single	123	37.2
Married	208	62.8
Unit worked		
Clinic	180	54.4
Outpatients’ department	68	20.5
Emergency	31	9.4
Intensive care	52	15.7
Work experience		
<5 years	98	29.6
6–10 years	73	22
11–20 years	89	26.9
>20 years	71	21.5
Working of own accord in the unit worked		
Yes	303	91.5
No	28	8.5
Status of willingly working in the nursing profession		
Yes	264	79.8
No	67	20.2
Status of receiving education on self‐compassion		
Yes	52	15.7
No	279	84.3
Total	331	100

### Nurses’ self‐compassion and caring behaviour levels

The nurses had a mean score of 3.50 ± 0.61 on the SCS. Examining the sub‐dimensions of the SCS, it was seen that they scored 3.25 ± 0.77 on self‐kindness, 2.23 ± 0.76 on self‐judgement, 3.30 ± 0.75 on common humanity, 2.33 ± 0.78 on isolation, 3.38 ± 0.75 on mindfulness and 2.35 ± 0.78 on over‐identification. On the CBI‐24, it was found that the nurses scored an average of 5.21 ± 0.56, while on the sub‐dimensions of the CBI‐24, they scored 5.20 ± 0.64 on assurance, 5.50 ± 0.58 on knowledge and skill, 5.13 ± 0.65 on respectfulness and 5.04 ± 0.68 on connectedness.

### Correlations between self‐compassion and caring behaviour

A positive correlation at a low level was found between the SCS total score and the CBI‐24 total score (r = 0.22, *p* < 0.01). A significant correlation was seen between all SCS sub‐dimensions and the CBI‐24 total score. It was observed that there were low‐level negative relationships between self‐judgement (*r *= −0.17, *p* < 0.01), isolation (*r* = −0.13, *p* < 0.05) and over‐identification (*r* = −0.14, *p* < 0.01) with CBI‐24. Additionally, low‐level positive relationships were found between self‐kindness (r = 0.20, *p* < 0.01), common humanity (*r *= 0.15, *p* < 0.01) and mindfulness (*r* = 0.26, *p* < 0.01) with CBI‐24. Furthermore, there were low‐level positive correlations between the SCS total score and the CBI‐24 sub‐dimensions (Table 2).

**TABLE 2:** Correlations between self‐compassion and caring behaviour (*n* = 331).

	CBI‐24				
SCS	Assurance	Knowledge and skill	Respectfulness	Connectedness	CBI total score
Self‐kindness	0.14^**^	0.18^**^	0.19^**^	0.23^**^	0.20^**^
Self‐judgement	−0.10	−0.23^**^	−0.15^**^	−0.13^*^	−0.17^**^
Common humanity	0.12^*^	0.15^**^	0.14^**^	0.15^**^	0.15^**^
Isolation	−0.08	−0.16^**^	−0.13^*^	−0.13^*^	−0.13^*^
Mindfulness	0.21^**^	0.23^**^	0.23^**^	0.26^**^	0.26^**^
Over‐identification	−0.09	−0.23^**^	−0.10	−0.11^*^	−0.14^**^
SCS total score	0.15^**^	0.25^**^	0.20^**^	0.21^**^	0.22^**^

*Note*: SCS, Self‐Compassion Scale; CBI‐24, Caring Behaviour Inventory‐24.

* *p* < 0.05;

** *p* < 0.01.

### Predictors of caring behaviour

According to the results of multiple linear regression analysis, working in intensive care units significantly predicted nurses’ caring behaviours in comparison with working in a clinic (β = −0.20, *p* < 0.01), the outpatients’ department (β = −0.18, *p* < 0.05) or the emergency service (β = −0.15, *p* < 0.05). Also, working willingly in the nursing profession (β = 0.23, *p* < 0.001) and the SCS sub‐dimension of mindfulness (β = 0.24, *p* < 0.05) were found to be significant predictors of caring behaviour (Table 3).

**TABLE 3:** Multiple linear regression analysis results regarding the prediction of CBI‐24 score.

Predictor	B	SE	β	t	p
Constant	111.57	7.50		14.86	<0.001^***^
Age					
20–29	2.10	4.12	0.07	0.51	0.610
30–39	−0.50	3.10	−0.01	−0.16	0.872
>40	Ref	Ref	Ref		
Gender					
Female	Ref	Ref	Ref		
Male	2.70	2.53	0.05	1.06	0.287
Educational level					
High school	−3.83	4.46	−0.05	−0.85	0.391
Associate degree	−1.23	4.11	−0.01	−0.30	0.764
Bachelor's degree	−0.39	2.53	−0.01	−0.16	0.869
Postgraduate degree	Ref	Ref	Ref		
Marital status					
Single	Ref	Ref	Ref		
Married	1.02	1.75	0.03	0.58	0.560
Unit worked					
Clinic	−5.64	2.06	−0.20	−2.73	0.007^**^
Outpatients’ department	−6.28	2.65	−0.18	−2.36	0.019^*^
Emergency	−7.38	2.99	−0.15	−2.46	0.014^*^
Intensive care	Ref	Ref	Ref		
Work experience					
<5 years	−1.81	4.53	−0.06	−0.40	0.689
6–10 years	−0.89	4.01	−0.02	−0.22	0.825
11–20 years	3.43	3.15	0.11	1.09	0.276
>20 years	Ref	Ref	Ref		
Working of own accord in the unit worked	3.55	2.62	0.07	1.35	0.177
Status of willingly working in the nursing profession	7.94	1.83	0.23	4.32	<0.001^***^
Status of receiving education on self‐compassion	2.45	2.04	0.06	1.19	0.232
SCS sub‐dimensions					
Self‐kindness	−0.21	0.34	−0.06	−0.62	0.530
Self‐judgement	−0.39	0.32	−0.11	−1.21	0.225
Common humanity	−0.08	0.34	−0.01	−0.23	0.811
Isolation	0.02	0.38	0.01	0.05	0.960
Mindfulness	1.08	0.42	0.24	2.54	0.011^*^
Over‐identification	0.17	0.38	0.04	0.44	0.655

*Note*: SCS, Self‐Compassion Scale; CBI‐24, Caring Behaviour Inventory‐24; B, unstandardised beta coefficients; S.E., standard error; β, standardised beta coefficients; *R* ^2^ = 0.194, adjusted *R* ^2^ = 0.137, *F* = 3.378, *p* < 0.001.

* *p* < 0.05;

** *p* < 0.01;

*** *p* < 0.001.

The model was statistically significant (*F* = 3.378, *p* < 0.001) and explained 19.4% of the variance of caring behaviour in nurses (adjusted *R* ^2^ = 13.7%). The nurses’ age, gender, work experience, education level, marital status, status of working in the unit where they worked of their own free will, receipt of education on self‐compassion and the SCS sub‐dimensions of self‐kindness, self‐judgement, common humanity, isolation, mindfulness and over‐identification did not predict significantly (Table 3).

## DISCUSSION

The aim of this study was to determine the levels of self‐compassion and caring behaviour in nurses, to investigate the relationship between self‐compassion and caring behaviour, and to determine the variables predicting caring behaviour. Previous studies investigating the correlation between self‐compassion and caring behaviour have been conducted with nursing students (Alquwez et al., 2021) and psychiatry nurses (Varghese, 2020). The present study was conducted with nurses working in different departments. Self‐compassion and caring behaviour in nurses are an important topic concerning all fields of nursing. Thus, this research will contribute to research and practice concerning self‐compassion and caring behaviour in nurses. The nurses in this study were found to have a medium level of self‐compassion and a high level of caring behaviour, and a significant correlation was found between self‐compassion and caring behaviour. Also, working in an intensive care unit, working willingly in the nursing profession and the self‐compassion sub‐dimension of mindfulness significantly predicted caring behaviour.

In this study, nurses’ self‐compassion scores were at a medium level. The nurses’ highest scores on the sub‐dimensions of the SCS were on the mindfulness sub‐dimension, and the lowest were on the sub‐dimension of self‐judgement. Similarly, in other studies, it was reported that nurses’ self‐compassion scores were at a medium level (Atharyan et al., 2018; Duarte et al., 2016; Malik et al., 2022). However, in the literature, no differences are seen in the scores obtained by nurses on the SCS sub‐dimensions. In some studies, the highest scores were obtained on the sub‐dimensions of mindfulness (Joy et al., 2023) or self‐kindness (Duarte et al., 2016), while the lowest scores were obtained on the sub‐dimensions of isolation (Duarte et al., 2016) or over‐identification (Atharyan et al., 2018). This may arise from the nurses’ individual, organisational or cultural differences (Hicdurmaz & Aydin, 2017; Yuksel et al., 2022). For example, in a qualitative study by Yuksel et al. (2022), nurses stated that the reasons for not showing self‐compassion were that managers had a job‐focused understanding, their workload was excessive, they saw self‐compassion as selfishness, and that they had a perfectionist personality. Also, studies show that self‐compassion in nurses increases self‐respect and coping skills (Joy et al., 2023) and reduces levels of depression and anxiety (Hategan et al., 2020). For this reason, when planning education on self‐compassion, nurses’ individual, cultural and organisational differences should be taken into account (Yuksel et al., 2022). This may increase the effectiveness of their education and better enable nurses to cope with stressful events (Cleare et al., 2019). Before planning education related to self‐compassion, it is recommended that a preliminary study be conducted to recognise the target group.

In this study, it was found that nurses’ caring behaviour scores were at a high level. When the subscales of the CBI‐24 scale were examined, it was found that nurses had the highest scores on the sub‐dimensions of knowledge and skill as well as assurance, and the lowest scores on respectfulness and connectedness. Similarly, it was found that in the literature, nurses’ caring behaviour scores were at a high level (Efil et al., 2022; Kibret et al., 2022). It was also found that the sub‐dimensions of connectedness and respectfulness had lower scores than knowledge and skill and assurance (Efil et al., 2022; Romero‐Martin et al., 2019). The sub‐dimensions of connectedness and respectfulness include behaviours such as being able to devote enough time to the patient, listening attentively to the patient, establishing empathy, behaving with patience towards the patient and including the patient in care. These behaviours are related to expressive or individualised caring (Wu et al., 2006). The reason for low connectedness and respectfulness scores may be that there are too few nurses in the clinics and that they give task‐oriented care (Efil et al., 2022). This is because the sub‐dimensions of knowledge and skill and assurance emphasise instrumental or duty‐focused care (Romero‐Martín et al., 2019). In task‐oriented care, nurses focus on fulfilling their responsibilities quickly and act according to the needs of the clinic rather than those of the patient. However, this can reduce individualised care (Fallon et al., 2018). Also, it is reported in the literature that patients perceive caring behaviour to be at a lower level than nurses perceive it (Flynn, 2016). Finding out patients’ expectations with regard to caring behaviour, supporting nurses’ physical and mental well‐being, increasing managers’ awareness of person‐specific care and designing a nursing education programme that covers all fields of caring may be important strategies in improving caring behaviour (Romero‐Martín et al., 2019). To promote clinics with an individualised care approach, it is recommended that collaborations between health policymakers and nurse managers be increased and that workshops be organised on this topic.

In this study, a correlation was found between levels of self‐compassion and caring behaviour in nurses. However, according to the results of multiple linear regression analysis, only the self‐compassion sub‐dimension of mindfulness significantly predicted caring behaviour. Different from this study, it was found in a study conducted with nursing students that caring behaviour was significantly predicted by the self‐compassion sub‐dimensions of self‐kindness and common humanity (Alquwez et al., 2021). This may arise from differences in the sample groups because self‐compassion and caring behaviours can be affected by the characteristics of the nurses such as their personality (Fradelos et al., 2022; Miley et al., 2024), emotional intelligence (Lewis et al., 2017; Şenyuva et al., 2014), empathy skill (Dogdu et al., 2022; Duarte et al., 2016) and exhaustion (Durkin et al., 2016; Romero‐Martin et al., 2019). It is recommended that in future studies, the correlation between self‐compassion and caring behaviour in nurses should be further researched and that the variables that could mediate this correlation should be determined.

Working in an intensive care unit and working willingly in the nursing profession were other variables predicting caring behaviour. Other studies showed similarly that nurses working in intensive care (Erol & Turk, 2019) and those working willingly in the nursing profession (Efil et al., 2022) had higher levels of caring behaviour. Nurses in intensive care units may form a stronger bond with patients for reasons such as the patients needing more care, the smaller number of patients per nurse and the nurses spending more time on caring (Lee & Seo, 2022). These factors may cause nurses to display caring behaviour at a higher level than in other units. Also, nurses working willingly in their profession experience less exhaustion and have more motivation to show caring behaviour (Efil et al., 2022; Uchmanowicz et al., 2020). For this reason, it is important to stress topics such as nursing philosophy and professional values frequently to be able to establish a strong link with the professions of nurses (Kaya et al., 2023). Nursing faculty members can do this by increasing the number of class hours on topics such as nursing philosophy and professional values. Moreover, increasing the use of technology in education can enhance students’ interest in these subjects.

According to the results of this study, nurses’ age, gender, work experience, education level, marital status, willingness to work in the unit worked, and their status of having received education on self‐compassion did not significantly predict caring behaviour. In the literature, it is seen that there were mixed results on this topic. In some studies, nurses’ age (Kibret et al., 2022), gender (Lee & Seo, 2022), work experience (Kibret et al., 2022), education status (Lee & Seo, 2022), marital status (Fallah et al., 2023) and working in a unit of their own free will (Erenoglu et al., 2019) were not correlated with caring behaviour, while in some studies male gender (Ilkafah et al., 2023), greater age and work experience (Arsat et al., 2023), being married (Lee & Seo, 2022), having a higher education level (Fradelos et al., 2022) and working in a unit which they themselves wanted (Çelik & Kin, 2022) were found to be correlated with caring behaviour. These mixed results in the literature may derive from some nursing education programmes not being care‐focused but preponderantly on a biomedical model (Romero‐Martin et al., 2019) and from a task‐oriented system being operated in clinics (Efil et al., 2022). Also, in this study, even though the levels of caring behaviour were higher in nurses who had received education on self‐compassion, no significant difference was found. This result may arise from too few structured lessons on self‐compassion and the small number of nurses who have received education on this topic (Andrews et al., 2020). It is recommended that structured lessons be planned in clinics on self‐compassion, that the concept of self‐compassion be included in nursing education programmes, and that nursing education programmes be based on a nursing model.

## LIMITATIONS

This study has various limitations. First, as it was a cross‐sectional study, causality cannot be established between the variables. Second, measurement instruments based on self‐reporting were used in the study. Measurement instruments based on self‐reporting can result in bias because participants tend to give responses that are more positive about themselves. Finally, because a convenience sampling method was used in this study, the generalizability of the results is limited. They cannot be generalised to other institutions or regions.

## CONCLUSION

The nurses’ self‐compassion levels were medium and their caring behaviour was at a high level, and their caring behaviour was higher in those who worked in intensive care, those who were working willingly in the nursing profession and in those with high scores on the self‐compassion sub‐dimension of mindfulness. The nurses’ age, gender, work experience, education level, marital status, status of working in a unit of their own free will and status of having received education on self‐compassion did not significantly predict their caring behaviour. In future studies, these variables should be taken into consideration and education programmes should be planned that would increase nurses’ levels of self‐compassion and develop caring behaviour. Also, there is a need for further research in different cultures and samples to clarify the correlation between self‐compassion and caring behaviour in nurses. Therefore, it is recommended that the structural equation model be used for the variables that could mediate the relationship between self‐compassion and caring behaviours, and that spiritual ways of influencing caring behaviours be explored using the grounded theory method.

## IMPLICATIONS FOR NURSING AND HEALTH POLICY

Our results demonstrate that the self‐compassion sub‐dimension of mindfulness is an important variable in improving care behaviour in nurses. Mindfulness means being aware of distressing life experiences and accepting them as they are. For an individual to be able to show self‐compassion, they first need to be aware of their own distressing life experiences. For this reason, mindfulness is an indispensable part of self‐compassion (Neff & Tirch, 2013). Increasing nurses’ mindfulness skills may enable them to improve their caring behaviour. This can be done through practices that support spirituality such as yoga, meditation, relaxation techniques, imagination, body scanning, meditative walking, non‐judgemental observation, body awareness and offering compassion to the body. For this reason, basic education in mindfulness should be planned in all departments, nursing managers should increase their awareness of this topic, and the concept of self‐compassion should be included in nursing education programmes.

## AUTHOR CONTRIBUTIONS

*Study design*: Arif Özparlak and Dudu Karakaya. *Data collection*: Arif Özparlak, Dudu Karakaya and Esra Çelik. *Data analysis*: Arif Özparlak and Hilal Kara. *Study supervision*: Dudu Karakaya. *Manuscript writing*: Arif Özparlak, Dudu Karakaya and Hilal Kara. *Critical revisions for important intellectual content*: Arif Özparlak, Dudu Karakaya, Hilal Kara and Esra Çelik.

## CONFLICT OF INTEREST STATEMENT

The authors declare that they have no conflict of interest.

## FUNDING INFORMATION

This research did not receive any specific funds from funding agencies in the public, commercial or not‐for‐profit sectors.

## ETHICS STATEMENT

The study was approved by the Akdeniz University Clinical Research Ethics Committee.
